# Medico-Chirurgical Transactions

**Published:** 1822-03-01

**Authors:** 


### XIII.

### *Medico-Chirurgical Transactions.* Vol. II. Part 2, with Plates.

Were we to say that the volume before us is equal, in interest or importance, to its earlier predecessors, we should certainly have the character of our judgment or veracity suspected. And as we do not deem it necessary to sacrifice this character for the sake of complimenting a public body, (which neither needs nor cares for compliments,) we shall candidly confess, that our disappointment and regret have, for some time, been excited by the "falling off" of transactions which, at one period, commanded the respect and called forth the admiration of Europe and America. It is perhaps needless to inquire into the cause or causes of this decadence, since we perceive it to be an inherent principle in every human institution or production, whether of the head or the hand. Is it very astonishing, then, that the periodical transactions of the Medico-Chirurgical Society should bow to that universal mandate of Nature which declares that—

> The seas shall waste, the skies in smoke decay,
> Rocks fall to dust, and mountains melt away!

It would be much more astonishing, indeed, if these transactions presented an exception which would be the only one in the annals of the world—and in nothing more strange than in the records of periodical publications on medicine.

As a bright side to this sombre picture we are happy to be able to state that, while the papers read at the Medico-Chirurgical Society have declined in value, the Society itself has risen and is still rising in numbers, respectability, and influence. It is also gratifying to add that, while the communicationss are not unfrequently of an inferior character, the discussions arising out of them very often assume the highest interest and importance.

We have one remark to add, before we proceed to our analytical labours. We imagine that the Society would do well to imitate the conduct of many societies on the Continent, by publishing *abstracts* of those papers which cannot be admitted, in their original forms, into the transactions. Many important hints and valuable facts would then be preserved for the profession at large—nor do we believe that the writers of these papers would feel displeased at the publication of their communications in a condensed form—on the contrary, we imagine they would prefer this to their entire suppression.

We shall make a point of giving, in future, a regular and rather detailed account of all papers appearing in these transactions; and we hope that on this, as on every other occasion, we shall carefully abstain from mixing private feelings (however they may be excited) with public duties—a practice dangerous to the writer, affronting to the reader, and inimical to the interests of a liberal and enlightened profession.

### I. *Cases of Bronchocele or Goitre, treated by Seton, with Observations, by* A. P. Copland Hutchison, Esq.

In the tenth vol. of the Medico-Chirurgical Transactions Dr. Quadri published a paper on the Treatment of Bronchocele by Setons, which induced Mr. Hutchison to adopt the practice. The case, which he selected for experiment, occurred in a woman, who had first perceived the disease thirteen years before. It appeared a few days after labour, which we have found a frequent cause of it, by producing a retardation in the reflux of the blood, and a consequent enlargement of the vessels of the neck. The tumour was of the size of an orange, and of rather a firm and hard structure. Mr. H. passed a long and narrow seton-needle armed with half a skein of silk-thread, obliquely through the substance of the gland, from the left lobe upwards, that being more enlarged, leaving a space of nearly two inches between the entrance and escape of the instrument. The trachea being pushed backwards upon the œsophagus, the trunks of the thyroid arteries escaped the needle, which, it must be observed, was carried through the gland midway between the integuments and the wind-pipe. No hæmorrhage of importance followed. At the end of a few days an erysipelas appeared, which was followed by a profuse discharge of thin acrid matter. The discharge from the wound was kept up by the occasional application of sabine-ointment; and at the end of three months the seton was removed, and another introduced from the lower part of the right lobe of the gland, so as to cross the course of the former. The latter seton having remained in its situation during two months, accidentally escaped; but it was not deemed necessary to replace it, as the discharge from and gradual reduction of the tumour continued afterwards to proceed in a favourable manner. A slight oozing of kind pus having been thus spontaneously supported for several months, about twelve from the time of the operation it subsided, and the disease was scarcely perceptible.

The seton seems to be better adapted to the soft and yielding species of bronchocele, than to that which is firmly indurated and lobulated. In the former, Mr. Hutchison says that the operator may boldly pass the instrument throughout the substance of the morbid mass with perfect safety. In the latter, it might be more prudent to perforate a smaller portion of the gland, and to repeat the operation on different parts of it in succession, in order to guard against inflammation in the mucous membrane of the trachea.

The deformity succeeding the cure has been found to disappear in great measure in a few months.

It has been asserted by Mr. Nerman, a student from Sweden, that in Denmark it is a common practice to make incisions into the substance of the diseased gland, and that bronchocele is thus frequently cured; but we are not informed whether the suppurative process is afterwards established or not. This disease has also been successfully treated by Dr. Coindet of Geneva, with solution or tincture of iodine; and Dr. Straub, of Howfyl, asserts, that this medicine may be advantageously used in all cases in which burnt sponge has been employed.

To the above case, in which the seton was employed, Mr. Hutchison adds one by Mr. Gunning, surgeon to St. George's Hospital, three by Mr. A. T. Thompson, and one by Mr. James, of the Devon and Exeter Hospital. In Mr. Gunning's case, eight days after the operation, the seton was removed, the parts contiguous to it being in a sloughing state; and a few days after the separation of the sloughs, the patient's health having gradually declined, she died after one of the severe attacks of tracheal irritation, to which she had been subject. The remaining portion of the thyroid gland was in a sloughing condition, and some slight streaks of inflammation were discovered in the lining membrane of the trachea after death.

In the first of Mr. Thompson's cases the operation was successful. A considerable discharge of blood followed immediately, and a slight one continued during two or three days. The seton was removed at the end of two months. The result of the practice in the second experiment was not known. The third case occurred in a man, aged sixty-one. The swelling was of the soft kind. The seton was withdrawn in three weeks, the tumour being greatly reduced.

Mr. Thompson was partly induced to try the effects of the seton, having witnessed the cure of a very large bronchocele by the application of a caustic, which occasioned the whole of the diseased gland to separate at the end of five months.

Mr. James's case was a most interesting one. The patient, a man aged 35, had received the best assistance which could be afforded him, without experiencing any benefit. The tumour was firm and elastic, and of the size of a large flattened orange. The progressive increase of it and the accompanying symptoms threatened his destruction. The seton was introduced through the centre of the swelling from the upper to the lower part. The next day, the obstruction returning and the face being flushed, he was bled and purged. The dyspnœa increasing, attended with distressing cough, suppression of the voice as in laryngitis, copious expectoration, haggard countenance, and a frequent, small, and weak pulse, the seton was taken out about ten days after the operation. At this time the patient expectorated transparent firm lymph, having the appearance of membrane; and the same substance could be extracted from the ulcerated orifices. An expectoration of puriform mucus was present, attended with orthopnœa. In less than two months the tumour entirely disappeared.

The practice of curing bronchocele by the seton, which was introduced by Foderé and others nearly half a century ago, appears until lately to have fallen into disuse. The attempts to revive it, which we have just detailed, have been sufficiently fortunate to justify us in recommending its adoption in all cases, in which the tumour is soft, and in others, where the symptoms portend a speedy dissolution, and the usual means have been found ineffectual. In young persons the operation will seldom be found requisite, because the sponge will in them usually effect a cure, and we have frequently observed the disease to disappear spontaneously before puberty. After this period, medicine is seldom of any avail, and our chief hope must rest upon local measures.

II. *Observations on the Scrofulous Inflammation of the Peritonæum occurring in Children, and frequently denominated Marasmus.* By George Gregory, M. D. Senior Physician of the St. George's and St. James's Dispensary.

Dr. Gregory thinks he has been able to distinguish three different states of abdominal disease in children, which have, as a common character, fever of a slow remitting kind, and emaciation.

"The *first* of these consists in simple disturbance of the functions of the intestinal canal without organic derangement." P. 261.

This, according to Dr. Pemberton, may prove fatal without inducing inflammation or ulceration within the abdomen, and constitutes the most common form of infantile remittent fever.

"The *second* form of marasmus is that in which the mucous membrane of the bowels is extensively implicated. After death ulcerations both of the great and small intestines are observed, with more or less enlargement of the mesenteric glands, and sometimes, though rarely, ulceration of them." 262.

In many instances Dr. Gregory presumes that this species is secondary, having been preceded by the one, which is found only to disturb the functions of the bowels.

The *third* form of marasmus is that to which Dr. G. wishes most particularly to attract our notice. He is induced to believe that it is primarily a disease of the peritonæum, and calls it the *scrofulous inflammation* of that membrane; on account of its being usually met with in scrofulous children. Disease of the mesenteric glands has not escaped his observation; but, having always seen it complicated with, or probably resulting from, a morbid state of the mucous or serous membrane of the abdomen, he views it in a subordinate light.

*Symptoms.* Gradually increasing tenderness of the abdomen, soon followed by paroxysms of acute pain, which grows more frequent and violent, and spreads from one part over the whole of the abdomen. The belly is at first enlarged and tense, and we have seen it completely tympanitic; but, as the disease advances, the swelling abates or subsides altogether, and is seldom perceptible after death. The pulse about 100; tongue generally clean; appetite irregular; thirst; intestinal discharges frequent, at first slimy and afterwards consisting of a whitish brown matter, and greatly exceeding in quantity the medicine and other ingesta. Emaciation, and in four or five months diarrhœa and petechiæ, which in three or four days destroy the patient. Strangury is a common symptom, and in several instances we have satisfactorily ascertained that there have existed a periodical increase of pain and a febrile exacerbation, for the most part after midnight.

*Necrotomy.* Dr. G's description of the morbid appearances on dissection exactly correspond with those, which we have ourselves witnessed.

" On cutting through the parietes of the abdomen, all trace of abdominal cavity will be wanting. The mesentery, bowels, and peritonæum lining the parietes, will be found united together into one mass. The peritonæum, in all its duplicatures, appears thickened; and, on cutting through the diseased mass, very large quantities of scrofulous matter will be found. The mucous membrane of the bowels, particularly (of) the small intestines, appears ulcerated in various places, and at these points of ulceration the convolutions of the intestines communicate, so that, instead of forming one line of canal, as they will continue to do even in advanced stages of common chronic peritonitis, they constitute a mass of tubes communicating freely with each other, and with the thickened and ulcerated peritonæal membrane by innumerable openings. The matter, which will be found both within and without the mucous membrane, will be observed to correspond exactly with that which was passing during life by stool." 266.

*Treatment.* Little impression having been produced on the disease by any medical treatment, Dr. G. has enumerated only a few remedies, and on these he appears to place no reliance. When laudanum becomes necessary in the latter period of the malady, he has not found it to produce constipation.

*Diagnosis.* Abdominal tenderness and paroxysms of pain, followed by the discharge of large quantities of thick white matter by stool, are considered by Dr. G. sufficient peculiarities to distinguish the scrofulous peritonæal inflammation from the other species of marasmus.

Dr. Gregory proposes to offer to the Society, on some future occasion, observations respecting hydrocephalus, when he will add his farther experience in the different species of marasmus. We shall be happy to give extended publicity to Dr. Gregory's remarks at all times.

III. *Case of Fractured Os Pubis successfully treated.* By HENRY COATES, Esq. Surgeon to the Salisbury Infirmary.

THE accident was occasioned by three persons falling with great force upon a woman from a coach, which was overturned. The fracture was readily discovered at the juncture of the ramus of the pubis with the ischium. The bladder, rectum, and the extremities retained their sensibility.

Mr. Coates procured a bandage of wide, woollen girth-web. This was drawn under the pelvis, and, by means of buckles and straps placed near together, was secured as tightly as the patient could bear. Two straps were attached to the back part of this belt, brought between the thighs and fastened in front; and pads were placed on each side the pubis. Bleedings, aperient and saline medicines were had recourse to, and at the end of five or six weeks the patient was able to walk without assistance, and recovered.

IV. *Case of Sudden Death, in which a Hydatid was found in the Substance of the Heart.* By DAVID PRICE, Esq. London.

THE boy, who was the subject of this case, was ten years old. He fell down suddenly and unexpectedly, and in a few minutes expired.

The contents of the cranium and of the abdomen were found perfectly healthy. A portion of the pericardium adhered to the heart, and in the muscular substance of the latter was discovered a large hydatid. Two ounces of dark-coloured fluid were floating in the pericardium.

V. *A Case of Aneurism of the Carotid Artery.* By HENRY COATES, Esq. Surgeon to the Salisbury Infirmary.

THE aneurismal tumour was seated in the left carotid, and measured five inches and a half in length, and four in depth. The patient, aged 41, was first a sawyer, afterwards a dragoon, and, lastly, a labourer in husbandry. This disease extended beyond the mastoid process, in part concealing the ear, and covering the edge of the inferior maxilla almost to the chin, where it became conical. The pulsation was strong, and ulceration had begun. The man complained of head-ache, had a slight cough, was confined to bed, had dyspnœa and dysphagia, and expectorated daily nearly three pints of mucus. The pupil of the left eye was contracted, and vision imperfect. He spoke indistinctly. The pulse 90.

Digitalis and venesection having been tried in vain, the operation of applying a ligature on the artery was deemed expedient. Only one ligature was employed, and the wound was drawn together with adhesive plaster. The patient became faint, and continued so for some minutes.

The next day the pulse having become hard and quick, he was bled from a large orifice, and other symptoms were relieved by aperients and sudorifics, and by opiates at bed-time; and about the eighth day the aneurismal mass had subsided to half its pristine size, and the contracted pupil had nearly recovered its natural dilatation and sensibility. On the 33d day the tumour was much enlarged and tender, and a blush of inflammation appeared on both sides of the neck, attended with much pain in the throat. The 34th day, the pulse being hard, venesection both local and general was had recourse to, and a fluctuation being evident, an opening was made, which gave vent to seven ounces of offensive blood and pus. On the 55th day six ounces of florid blood were suddenly discharged from the aneurismal sac, after which the hæmorrhage spontaneously ceased; and the pulse remaining hard, bleeding from the arm was again repeated. The tonsils were then inflamed. From this to the 63d day hæmorrhage repeatedly appeared; and about the 67th day the patient fell into a state of collapse with dysphagia, singultus, and anxiety, and in the evening expired.

*Necrotomy.* The ligature had been applied to the artery at the distance of an inch and a half from its origin. The vessel itself was impervious for the space of an inch. An artery, which admitted a probe, was discovered extending from the lower jaw into the sac. The back part of the aneurismal bag adhered to the bodies of four of the vertebræ.

Mr. Coates is inclined to believe that the event of this case might have been more fortunate, had the contents of the tumour been evacuated by art, before the inflammation had made its appearance.

VI. *Case of Malformation of the Heart.* By George Gregory, M. D. Senior Physician to the St. George's and St. James's Dispensary.

The patient was from birth of a blue colour, thin, and subject to constant dyspnœa; and at the age of eighteen years he died of pulmonary consumption.

The preternatural appearances in the heart were the following: the aorta and pulmonary artery arose from the right ventricle; the septum ventriculorum was deficient at its base; the opening in the septum corresponded exactly with the situation of the aorta at its origin, and effected a communication between the ventricles; the pulmonary artery was but little smaller than usual.

VII. *A Case of Chorea successfully treated by Arsenic.* By George Gregory, M. D. Senior Physician to the St. George's and St. James's Dispensary.

The Species of Chorea, for which arsenic has been successfully exhibited, is *said to be* unaccompanied with disorder in the stomach and bowels.

Dr. Gregory's patient was seven years old, and had suffered with the disease above six weeks. Several remedies had been tried in vain. She appears to have taken liquor arsenicalis about sixteen days, beginning with three drops, and gradually increasing the dose to seven or eight. The medicine twice produced sickness and was suspended, and in about three weeks the girl was discharged cured.

For the use of so active a medicine as arsenic, we are astonished that minims are not preferred by Dr. G. to drops, which vary according to the size and shape of the aperture through which they pass. Indeed on all occasions we think the minim-measure ought to supersede the use of drops.

We have seen several cases of chorea, in which the digestive organs have not appeared to be affected, but, after a careful examination of the intestinal discharges, we have found reason to believe that the liver has been torpid: the fæces having been well formed, but not exhibiting that complexion, which is the result of a due admixture of healthy bile. Whether the hepatic disorder may have been primary or secondary, we cannot determine; but we have, in these cases, observed such a debility in the mental powers, as to occasion temporary idiotism. Both the mental and physical infirmities have however always been removed by the exhibition of a grain or two of calomel, repeated twice or three times a day, until the stools have a natural aspect, and the circulation, which was before slow and interrupted, has been accelerated for a sufficient length of time to insure a wholesome energy in the viscera, which were oppressed.

### VIII. *On the Efficacy of the Bark of the Pomegranate-Tree in Cases of Tænia.* By P. Breton, Esq. Surgeon to the Rhamgur-Battalion in the East Indies.

Eight cases of tænia are related, in four of which a decoction of pomegranate was employed, and in the others the powder, with equally good effect. The decoction was prepared by boiling two ounces of the dried bark in a pint and a half of water, until it was reduced to twelve ounces. Of this two ounces were given every half hour, until four or five doses were consumed; when vertigo, sickness, and uneasiness in the bowels commenced. The medicine was then suspended, and at some time within twelve hours the worm was expelled. The powder was administered in the dose of a scruple to a boy, and two scruples to an adult, every half hour, for five or six times, when the same symptoms and effects resulted, as when the decoction was exhibited.

### IX. *On the Efficacy of the Bark of the Swietenia Febrifuga, as a Substitute for that of the Cinchona.* By P. Breton, Esq.

From the resemblance of the extract of the swietenia to gum kino, and the predominance of its astringent quality, it is probable that its effects will be found analogous to those of any other vegetable astringent. The cases which are brought forward, and the praises bestowed upon it, by no means satisfy us that it possesses any specific property in the cure of disease, besides what might be expected to result from its astringency. The pathology of fever is now so much better understood, than it was when cinchona was first introduced, that few modern practitioners will, we apprehend, be solicitous to obtain any substitute for a medicine which is likely soon to be nearly exploded.

X. *On the Physiology of the Ear.* By Joseph Swan, Esq. Lincoln.

The Medico-Chirurgical Society some time ago published a paper of Mr. Swan's respecting the physiology and pathology of the ear, in which he presumed that people born deaf and dumb, and who had no defect in the auditory nerves, might be made to hear through the medium of the facial nerves, and thus have their unfortunate situation amended. To substantiate this opinion, he has in the present volume adduced a case, in which the external passage to the ear was imperforate, and sounds were heard through the nerves on the face. The person can also speak intelligibly.

Mr. Swan thinks that the reason why those who are born deaf are not more frequently able to acquire a degree of perfection in hearing, is because their whole attention is apt to be taken up with signs, and no methods have been generally used to increase the power of the provision usually made by Nature for supplying the defects occasioned by imperfections of the tympanum.

"Presuming that what I have said is well founded, it is not reasonable to expect that the powers of the facial nerves should ever be fully developed in dumb children, if their instructors do not direct the whole, or by far the greater part of their attention to the proper exercise of these nerves. And if this is to be done effectually, it must probably first be by the assistance of instruments, to increase the effect of sound; and when these have been properly used, and have answered the intended purpose, then by gradually lessening their power until common sounds can be heard." 336.

XI. *Case of Amputation of Part of the Tarsus and Metatarsus, and Preservation of the Shape and Usefulness of the Foot.* By John Dunn, Esq. Scarborough.

A boy had suffered more than two years with caries in the bones of the tarsus attended with hectic fever. A tourniquet being applied to the thigh, Mr. Dunn cut through the extensor-tendons of the foot, and removed the os cuboides and the external cuneiform bone. No serious hæmorrhage followed. An improvement having taken place in the boy's health, about three weeks afterwards the two other cuneiform bones, and the os naviculare were dissected out, and the diseased tarsal ends of the metatarsal bones of the second and third inner toes were sawed off. A profuse hæmorrhage and syncope followed. A part of the astragulus in front being diseased, was scraped away, and the hæmorrhage was restrained by dry lint and a roller. On the third day after this second operation, the bleeding returned, and the wounded vessel was secured. Sinuses made their appearance, but the foot was quite healed in two months by means of adhesive straps. The boy now walks without any pain or apparent lameness, and the natural appearance of the foot is but little altered, excepting that it is an inch and a half shorter than the other.

Remarks on this case are subjoined by Mr. Copland Hutchison, in which he mentions an instance, where he removed several bones from the tarsus, after which his patient recovered with the use of his foot.

XII. *An Account of a Case in which numerous Calculi were extracted from the Urinary Bladder, without the Employment of Cutting Instruments.* By Sir Astley Cooper, Bart. F. R. S.

"When a great number of calculi are found in the bladder, as was the case in the Rev. Mr. Bullen, the circumstance is generally attended with an enlargement of the prostate gland, and it depends upon a sacculus being formed in the bladder directly behind the enlarged gland. In these cases the bladder is rarely completely emptied of its contents, and the calculi crystallize from the urine retained in this sac.

"Such stones do not in general acquire the magnitude of those formed under the usual circumstances, and, from their number and collision against each other, their surfaces are generally smooth, and their shape is commonly rounded." 357, 358.

"When calculi are thus placed, they are so concealed in the bag in which they are contained, that, in sounding, the instrument is liable to pass over them without their being discovered, and it is therefore necessary to dip the point of the sound towards the rectum as it enters the bladder, in order to detect them, or to pass the finger into that intestine, to raise them from the bed in which they are concealed; and it is for want of attention to this circumstance that I have known a person pronounced not to have the stone, from whom I afterwards removed thirty-seven by the operation of lithotomy." P. 358.

The instrument, which Sir Astley made use of in the case, which he has published, was in the shape of a sound, consisting of two blades, that acted like a pair of forceps in grasping the stones.

"Mr. Bullen was placed across his bed, with his feet resting on the floor, and a silver catheter was then introduced, and the bladder emptied of its urine. I then passed the forceps into the bladder, and was so fortunate in my first operation as to extract eight calculi.

The instrument gave but little pain on its introduction, but when opened to its greatest extent, and the stones admitted between its blades, their removal was painful, more especially at the glans penis, which appears to be the portion of the urethra, which furnishes the greatest resistance to the removal of the stones." 359.

A dose of opium was given after each operation, which was repeated seven times before the calculi were all removed.

We have had opportunities of seeing this distinguished surgeon make use of this instrument, which appears an admirable invention, and perhaps capable of still farther improvement.

### XIII. *On Sloughing Phagedæna.* By Richard Wilbank, Esq.

Mr. Wilrank has generally noticed this disease in the lowest class of prostitutes. It begins in the form of an irritable boil, elevated and surrounded by a dusky inflammation, seated generally in the clift of the nates, in the groin or upper part of the thigh. The apex of the boil vesicates, and bursting exposes a stratum of adherent straw-coloured flocculi, with a viscid secretion resembling pus, which is interspersed with darker points of a brown or grey tint. The surface enlarging, the centre becomes depressed, and the oval or circular sore is surrounded by a circumscribed thickening and an increasing halo of dark inflammation. From the darker points of the ulcer hæmorrhage occurs at times, and a peculiar fetid odour is observable. As the putrescence of the slough advances, much foul secretion appears, and shreds of pulpy matter are detached by the removal of the dressings. The sloughs are grey or dark brown. The pain becomes constant and agonizing, and the patient is found lying on her back with the thighs separated and bent upwards. The disease now spreads with rapid strides and is highly contagious. At length the constitution participates in the mischief which is going on: restlessness, loss of appetite, furred tongue, anxiety, pains in various parts of the head, accelerated pulse, hot skin, thirst, bilious vomiting and purging present themselves. Delirium rarely supervenes.

*Treatment.* Mercury has been constantly found exceedingly hurtful. Bleeding, particularly in plethoric habits, is proper in the early stage; but leeches are objectionable on account of the tendency of their bites to assume the morbid action of the adjoining parts. Large doses of opium at regular intervals are serviceable, particularly where the stomach is irritable. Bark is generally contra-indicated by the disposition to diarrhœa. The local means on which indeed Mr. W. places his principal reliance, both for relief and cure, consist in the application of nitric acid and other stimulating dressings.

"The method from which I have derived most benefit is as follows: if the disease be not far advanced, I at once apply the undilute acid, after cleansing the surface with tepid water, and absorbing the moisture with lint. Where, however, there is a thick and pulpy slough, it is better to remove as much as possible with forceps and scissars, before the application is made. The surrounding parts being then protected with a thick coating of lard or cerate, I proceed to press steadily and for some minutes a thick pledget of lint, previously immersed in the undilute acid, on every point.

XIV. *An Account of a Case of Tetanus successfully treated in the York Military Hospital at Chelsea.* By M. A. Burmester, Esq.

This was a case of traumatic tetanus preceded by fever, from exposure to cold and moisture. It was treated by copious depletion, opium, mercury, and the *warm* bath; and after ptyalism had been produced, the patient was directed to take the compound powder of ipecacuanha.

Another case is mentioned in which an unexpected recovery happened apparently from the supervention of gangrene in the wound, after salivation had been established, and opium and the warm bath had been employed, without any visible advantage.

XV. *Case of a Separation of a Portion of the Uterus during severe Labour.* By P. N. Scott, Esq. Norwich.

During a tedious and very severe labour Mrs. Hall felt the uterus suddenly snap. The pains instantly ceased, and uterine hæmorrhage came on attended with syncope, cold perspiration, feeble pulse, and vomitiug of a brownish fluid. On introducing his hand Mr. Scott found among the coagula a portion of the uterus, containing the os uteri and an irregular part of the cervix surrounding it. The delivery was completed with the vectis. For four or five days it was necessary to evacuate the bladder with the catheter, distention of the abdomen with fever followed, and the bowels became exceedingly costive; but the patient at length got well. She has a slight prolapsus uteri, and menstruates.

XVI. *A Case of Inguinal Aneurism successfully treated by tying the external Iliac Artery.* By Edward Salmon, Esq. Surgeon to the First Battalion of the Third Regiment of Guards.

The aneurism had existed ten months, and extended to Poupart's ligament. An incision was made through the integuments three inches and a half in length from the spine of the ilium to the face of the tumour. The aponeurosis of the external oblique was divided to the same extent. The internal oblique and the transverse muscles were divided with a probe-pointed bistoury to the extent of one inch and a half. The peritonæum being pushed aside, an incision was made with the scalpel on each side of the iliac artery, and a ligature was carried under it and tied. The wound was brought together with a suture and adhesive plasters. No difference in the temperature of the extremities followed, and, on the third day, the wound had united. On the seventh day, the wound was disunited by a discharge of sanies and pus. Three weeks after the operation, the ligature came away, and the wound had nearly healed; and at the end of two months, the man was discharged in good health, with a free use of his limb, and scarcely any tumour.

---

### XVII. *On Lithotomy.* By Philip M. Martineau, Esq. Senior Surgeon to the Norfolk and Norwich Hospital.

Mr. Martineau is an advocate for the lateral operation, and attempts to prove that Mr. Carpue's reasons for preferring the high operation are by no means conclusive.

In the first years of his practice, Mr. M. was not very successful, and, finding that his misfortunes arose from the use of the cutting gorget, he laid it aside. During the last seventeen years, he has employed the knife only, and the blunt gorget as a conductor for the forceps; and in this period, he has only lost two, out of eighty-four patients. He subjoins a table, shewing the age, name and event of every case. His first incision is made nearly in a line with the raphé, and the staff he uses, has a groove much wider and deeper than is commonly made. Feeling the groove of the staff, he introduces the point of the knife into it, as low down as he can, and cuts the membranous part of the urethra, continuing the knife through the prostate into the bladder. Instead of enlarging the wound downwards, he turns the edge of the blade towards the ischium, and makes a lateral enlargement of the wound in withdrawing the knife. He takes the *staff into his left hand*, while he introduces the blunt gorget with his right; and, after the latter is within the bladder, he introduces his finger, and endeavours to feel the situation of the stone. He always uses the straight forceps, and says it will be found more easy to extract a stone whole by rather large forceps, than with flat or small ones.

After the operation, a piece of lint is laid *over* the wound, and the air is excluded as much as possible, by a pledget of tow: it being Mr. M's object, to heal by the first intention. Coagula are removed by the finger pushed through the opening into the bladder, or by a female catheter. Pain should be relieved by opium, and abdominal tension by fomentations, blisters, and aperients. Mr. M. *never bleeds* his patients after lithotomy, and observes that, he has witnessed such debility succeed venesection, as could not be overcome; and he considers leeches altogether useless. The diet for the first two or three days, is mild, and, if fever and inflammation are absent, it is afterwards more generous. In general, Mr. M. believes, that death is oftener produced by exhaustion and despondency, than by acute disease.

In Norfolk and Suffolk, stone in the bladder is almost exclusively confined to the poor, and it appears often in infants, before diet can have any influence. The food of the poor is neither bad nor sparing, and the people are remarkable for cleanliness. It is a curious fact, that after the operation, scarcely a case occurs, in which the stone is generated a second time; and when that does happen, it may usually be traced to the breaking of the stone, whereby a fragment is left behind as a future nucleus.

XVIII. *Case of Cynanche Laryngea, in which Tracheotomy and Mercury were successfully employed: with Remarks.* By William Porter, Esq. A. M. Surgeon to the Meath Hospital and County of Dublin Infirmary, and to the Dublin General Dispensary.

The patient was strong and about thirty years old.

*Symptoms.* The face pale and swollen, and the lips livid:

"He sat with his mouth closed, but his nostrils widely extended; his eyes seemed protruded and starting from their sockets, but at the same time the conjunctiva appeared very white, and covered with a watery suffusion. There was altogether an expression of indescribable anxiety in his countenance. His pulse was hurried but not irregular; his breathing very laborious; he made two, three, or even more attempts at inspiration for one expiration, and his muscular heavings and convulsive struggles for breath were truly painful to behold. He breathed with a peculiar hissing or whistling sound, giving a distinct idea of the forcible passage of air through a contracted aperture, and he had almost lost his voice, the utmost endeavour at speech amounting only to an indistinct whisper." 416.

*Treatment.* Ten grains of calomel. The veins of both arms opened at once, and thirty or forty ounces of blood withdrawn, the patient being erect. Two hours after this the pulse was scarcely to be felt, the extremities were cold, and he was almost insensible. While in this state tracheotomy was thought necessary.

"An incision was made nearly three inches in length, commencing a little above the cricoid cartilage, and continued towards the sternum, dividing the skin and cellular substance down to the muscles. At this period of the operation two small lymphatic glands were exposed, which protruded forwards, and interrupting the view of the parts, were cut away. The incision was then carried deeper, still preserving the exact central line of the neck, until a fascia covering the trachea was exposed; and here lay the greatest difficulty of the operation. The trachea was moved upwards and downwards behind this fascia, according to the patient's exertions to breathe, and it was impossible to open it satisfactorily until this membrane was completely removed, a proceeding that occupied some time: it was however effected; the trachea was laid bare, in extent about three-fourths of an inch, and a circular portion removed, the diameter of which might have been nearly one-fourth of an inch." 419.

The moment the air was admitted through the wound into the trachea, the patient felt immediate relief, and began to recover his sensibility. A silver tube was passed into the aperture and retained by means of tape. Although the central slip, connecting the thyroid lobes, was completely divided, there was scarcely any bleeding. One large thyroid vein is sometimes found running along the middle of the trachea, which should always be carefully avoided.

Two more ten-grain doses of calomel were given at intervals after the operation, and warm wine and water allowed. The second day, the canula having slipped out, the symptoms returned, but were speedily relieved. Three more ten-grain doses of calomel were taken, and on the third day two doses of the same strength, although the bowels were open. On the fourth day salivation commenced, and the man made a good attempt to speak; and on the fifth, the opening into the trachea being found too small, it was enlarged, so as to render it three-eighths of an inch long. From this time nothing particular occurred to retard his recovery; and on the 21st day after the operation the wound in the trachea had united, and the patient was soon afterwards discharged in perfect health.

When inflammation in the larynx assumes an acute form, Mr. Porter conceives that it is probable an effusion of fluid may take place in the submucous structure in such quantity, and with such rapidity, as to close up the passage and produce suffocation.

" A melancholy proof of this lately occurred in the person of a young gentleman of high attainments; he only complained during the day of sore throat, and on the morning following was found in his bed quite dead. On dissection, the rima glottidis was found completely closed by the œdematous swelling of the mucous membrane." 428.

As soon as the nature of the disease is ascertained, and it is found to be of an active nature, Mr. P. advises as early an operation as possible, with the view of preventing the formation of disease in the lungs, as well as of affording speedy relief to the sufferings of the patient.

" If, then, the disease has occurred suddenly, and its symptoms have attained an alarming height in a short space of time, if there is expressive difficulty of breathing, and laborious muscular efforts to carry it on, the practitioner has but one resource, and that one will be in an operation. And although it is very possible that even in this he may not prove successful, if diseased action has already been formed in the lungs, yet he will be justified in the attempt, from the consideration that it is the only hope of safety he can hold out to his patient, and he will, at all events, be in some degree rewarded by the immediate alleviation it will afford to the most distressing symptoms." 437.

In the chronic as well as in the acute form of the disease there is no medicine, which has yet been found to possess such efficacy as mercury, when administered in such quantity and in such a manner as will rapidly bring the constitution under its influence; and in these cases, should any sudden or severe exacerbations threaten the lungs with disease, bronchotomy may be safely resorted to, and will probably be followed by a favourable result. It is an unfortunate circumstance, however, that the diagnosis of laryngeal inflammation is rendered difficult by the similarity of symptoms produced by different affections of the larynx.

We have thus attempted an outline of Mr. Porter's excellent paper. It is, however, so full of useful information on the subject of laryngitis, that we think our readers will be much gratified and instructed by an attentive perusal of the original publication in the Society's Transactions.

XIX. *Case of a large, adipose Tumour successfully extirpated.* By Sir Astley Cooper, Bart. F.R.S. Surgeon to Guy's Hospital.

Nicholas Pearson, aged 57, was admitted into Guy's Hospital, with an adipose tumour on the abdomen, which had been growing about forty years, and was at first no larger than a pea. Its dimensions at the time of his admission were prodigious:

"Measuring one yard and a quarter around its greatest circumference, and eighteen inches around its neck, extending, when he was sitting down, to his knees: it had increased most rapidly during the last three years, but up to the time of his admission, he expressed no other inconvenience than that of the weight he had to support, which of itself rendered him so perfectly incapable of obtaining his bread, that he was driven to the necessity of its removal." P. 443.

*Operation.*—"The first step of the operation was to draw the tumour to the patient's right side, and then to make an incision through the integuments and cellular membrane at its base; separating the swelling so far from its connexions as to be enabled to ascertain that it was not connected with hernia, or in any way with the abdomen; but in this investigation it was found, that a considerable portion, much more sensitive than the rest of the tumor, did project from the swelling into the umbilicus, but that it was not a hernia. Having ascertained this important point, the remaining part of the operation consisted in a simple dissection, with the application of ligatures to the veins, which were of considerable size, and bled freely, and to the arteries, which, considering the bulk of the tumor, were not so much enlarged as might have been reasonably expected. The patient lost but an inconsiderable quantity of blood during the operation." 443.

The weight of the tumour, independently of the blood it had contained, was 37℔. 10 oz.

No bad symptoms followed, and the wound healed principally by granulations. In eight days the man was able to walk in his ward.

APPENDIX I. *Abstract of the Account of a Case of Adhesion of the Labia Pudendi in a Negro, obstructing Delivery, drawn up by* Dr. WILLIAM RUSSEL, of Jamaica: *presented to the Society by* WILLIAM ROOTS, Esq. Surgeon of Kingston on Thames; *dated, Cascade, St. Mary, Jamaica, June* 12, 1819.

APPENDIX II. *Account of a Child of three Years of Age, in whom there appeared Signs of Puberty. Abstracted from a Paper communicated to the Society by* GILBERT BRESCHET, M.D. Superintendent of the Anatomical Department of the Faculty of Medicine in Paris; *dated, December*, 1820.

A boy, between three and four years old, with the same signs of premature puberty, is now exhibiting in London.

